# BCATc modulates crosstalk between the PI3K/Akt and the Ras/ERK pathway regulating proliferation in triple negative breast cancer

**DOI:** 10.18632/oncotarget.27607

**Published:** 2020-05-26

**Authors:** Mai Ahmed Shafei, Thomas Forshaw, Jasmine Davis, Arwa Flemban, David Qualtrough, Sarah Dean, Claire Perks, Ming Dong, Robert Newman, Myra Elizabeth Conway

**Affiliations:** ^1^Faculty of Health and Applied Sciences, University of the West of England, Coldharbor Lane, Bristol, UK; ^2^IGFs and Metabolic Endocrinology Group, University of Bristol, Bristol Medical School, Bristol, UK; ^3^Department of Chemistry, North Carolina Agricultural and Technical State University, Greensboro, NC, USA; ^4^Department of Biology, North Carolina Agricultural and Technical State University, Greensboro, NC, USA

**Keywords:** BCAT, PI3K-AKT, ERK, breast cancer

## Abstract

The cytosolic branched chain aminotransferase (BCATc) protein has been found to be highly expressed in breast cancer subtypes, including triple negative breast cancer (TNBC), compared with normal breast tissue. The catabolism of branched-chain amino acids (BCAAs) by BCATc leads to the production of glutamate and key metabolites which further drive the TCA cycle, important for cellular metabolism and growth. Upregulation of BCATc has been associated with increased cell proliferation, cell cycle progression and metastasis in several malignancies including breast, gliomas, ovarian and colorectal cancer but the underlying mechanisms are unclear. As nutrient levels of BCAAs, substrates of BCATc, regulate the PI3K/Akt pathway we hypothesized that increased expression of BCATc would contribute to tumour cell growth through upregulation of the insulin/IGF-1 signalling pathway. This pathway is known to potentiate proliferation and metastasis of malignant cells through the activation of PI3K/Akt and the RAS/ERK signalling cascades. Here we show that knockdown of BCATc significantly reduced insulin and IGF-1-mediated proliferation, migration and invasion of TNBC cells. An analysis of this pathway showed that when overexpressed BCATc regulates proliferation through the PI3K/Akt axis, whilst simultaneously attenuating the Ras/Erk pathway indicating that BCATc acts as a conduit between these two pathways. This ultimately led to an increase in FOXO3a, a key regulator of cell proliferation and Nrf2, which mediates redox homeostasis. Together this data indicates that BCATc regulates TNBC cell proliferation, migration and invasion through the IGF-1/insulin PI3K/Akt pathway, culminating in the upregulation of FOXO3a and Nrf2, pointing to a novel therapeutic target for breast cancer treatment.

## INTRODUCTION

Triple negative breast cancer (TNBC) is the most aggressive subtype of breast cancer and is associated with a poor prognosis [1]. TNBC is defined by the absence of oestrogen and progesterone receptors and the absence of HER2 overexpression. There is a lack of targeted therapy for TNBC due to wide heterogeneity for this subtype, which contributes to increased drug resistance, virulence and difficult to treat nature [2]. A key event promoting tumour cell proliferation and migration is metabolic reprogramming, where in response to oncogenic signals increased nutrient supplies drive cancer progression [3]. Several pathways have been associated with tumour progression including the phosphoinositide-3 kinase (PI3K)/serine/threonine-specific protein kinase (Akt) and the Ras/extracellular-signal-related kinase (ERK) pathway (also known as the Ras-Raf-MEK-ERK pathway). These pathways are controlled through the import of nutrients into cells by growth factors, such as insulin-like growth factor 1 (IGF-1), epidermal growth factor (EGF) and insulin, which mediate a cascade of events that regulate numerous cellular processes including cell growth and proliferation [4]. Upregulation of key signalling proteins of these pathways such as PI3K and ERK have been linked to several cancers including lung, thyroid and pancreatic cancer [5].

More recently the branched chain aminotransferase proteins (BCAT), which catalyse the transamination of the branched chain amino acids (BCAA), leucine, valine and isoleucine to their respective α-keto acids and glutamate (reviewed in [6]) have been identified as regulators of cell proliferation and migration [7, 8]. This cytosolic protein, BCATc, regulated by c-Myc, was found to be upregulated in a variety of cancers including gliomas [7, 9] ovarian, colorectal and breast [8, 10, 11]. The BCAA, particularly leucine, are potent nutrient signals and together with growth factors such as the IGFs and the hormone insulin regulate PI3K/Akt signalling [12]. Nutrient status is also sensed by the general control non-derepressible 2 kinase (GCN2) co-ordinates cell growth, proliferation and cell survival [13]. Therefore, by upregulating membrane transporters and nutrient uptake, cancer cells support the increased demand for macromolecules needed for cell growth [14].

Emerging evidence has indicated that the BCAT proteins, traditionally assigned metabolic roles, may have additional ‘moonlighting’ cellular functions, which are regulated through their peroxide sensitive-redox active CXXC motif [15]. We have recently established that the BCAT proteins can form thiol-dependent interactions with key signalling molecules such as those in the PI3K/Akt and the Ras/ERK pathways (unpublished observations) and show that phosphorylation of BCAT by PKC kinases is redox-dependent [16]. We showed that BCATc regulated autophagy through an interdependence of redox-regulated phosphorylation, supporting a ‘moonlighting’ role for this metabolic protein.

In this study, we show that BCATc regulates cross-talk between the PI3K/Akt and the Ras/ERK pathway. Using the TNBC cell model MDA-MB-231, we demonstrate that knockdown of BCATc results in a significant decrease of IGF-1 and insulin-mediated cell proliferation and migration. Subsequent analysis demonstrated that cell proliferation was associated with activation of the PI3K/Akt pathway that resulted in increased activation of FOXO3a, a transcription factor known to be involved in cell proliferation. Importantly, BCATc at the same time suppressed phosphorylation of ERK, indicating that regulation of proliferation through BCATc is primarly through the PI3K/Akt pathway rather than through ERK signalling, thus highlighting the plasticity of tumours to advance and adapt to changing environments.

## RESULTS AND DISCUSSION

### BCATc regulates proliferation, migration and invasion through the IGF-1 and insulin signalling pathway

**Figure 1:**
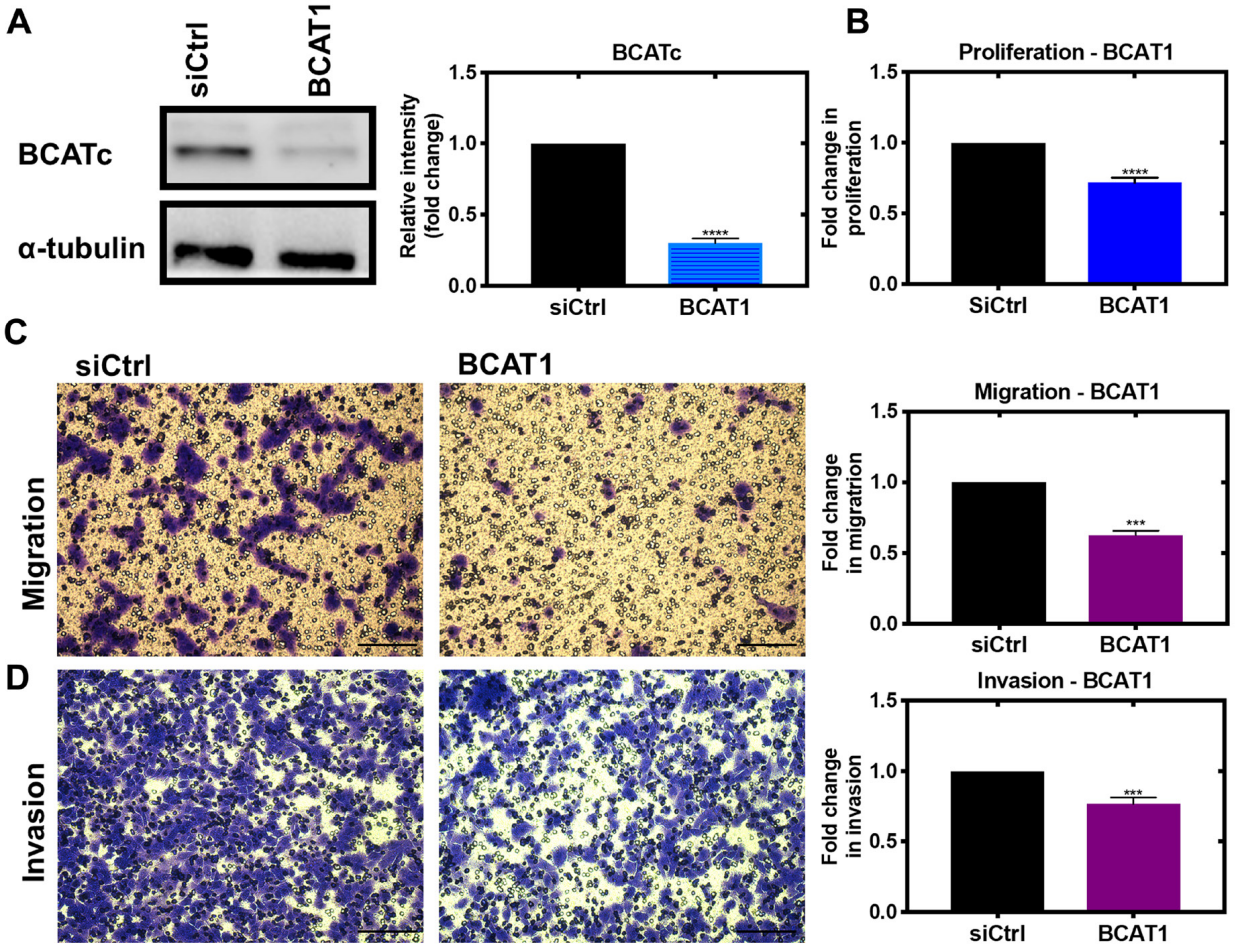
Knockdown of *BCAT1* significantly reduces proliferation, migration and invasion of MDA-MB-231 cells. Cells were treated with 20 nM *BCAT1* siRNA for 72 hours and the effect on proliferation assessed using the tritiated thymidine incorporation assay, migration was assessed using cells seeded onto 8 μm Transwell inserts (Greiner Bio-One) coated with collagen and after 24 hours, migrated were fixed and stained with 0.2% Crystal Violet, solubilised and absorbance measured and to assess invasion Matrigel added to the inserts as described above (**A**) Respective densitometric analysis of fold changes of protein expression relative to ɑ-tubulin are presented to the right of immunoblots. (**B**) Fold change in disintegrations per minute (DPM) and representative images of (**C**) migrated cells and (**D**) invaded cells with fold changes in absorbance at 590 nm ± SEM presented (*n* = 3) ^***^ *p* < 0.001 and ^****^ *p* < 0.0001 (scale bars = 100 μm).

Using siRNA we show that BCATc is intrinsically involved in mediating cell proliferation, migration and invasion of MDA-MB-231 cells (Figure 1A–1D). Similar studies have also reported a decrease in proliferation, metastasis and invasion of tumour cells in breast, colon and hepatocellular cancer in response to *BCAT1* knockdown [8, 11, 17]. The oncogene c-Myc not only upregulates *BCAT1* but transporters associated with glutamine and the neutral amino acid transporter 5 (SN5) [18]. Accumulation of glutamine and upregulation of glutaminase, which converts glutamine to glutamate, enhances glutathione synthesis, TCA cycle activation together with lipid and protein synthesis promoting cell growth and proliferation [19]. Moreover, leucine (a key substrate for BCAT), when restricted, has been shown to reduce cell proliferation, in several cancer cell lines including malignant melanoma (A375), lung cancer (A549), ovarian cancer (A2780) and breast cancer (MCF-7 and MDA-MB-231) [20] supporting a role for BCAA metabolism in regulating cell proliferation. Leucine together with glutamine is required for the activation of mTOR, as it relies on glutamine export for the intracellular transport of leucine through the bidirectional SLC7A5/SLC3A2 transporter [21]. In acute lymphoblastic leukaemia (ALL) deletion of *SLC7A5* (a high affinity transporter for glutamine) impaired T-cell tumour progression suggesting that several aspects of BCAA metabolism are important in regulating cell proliferation [22].

**Figure 2:**
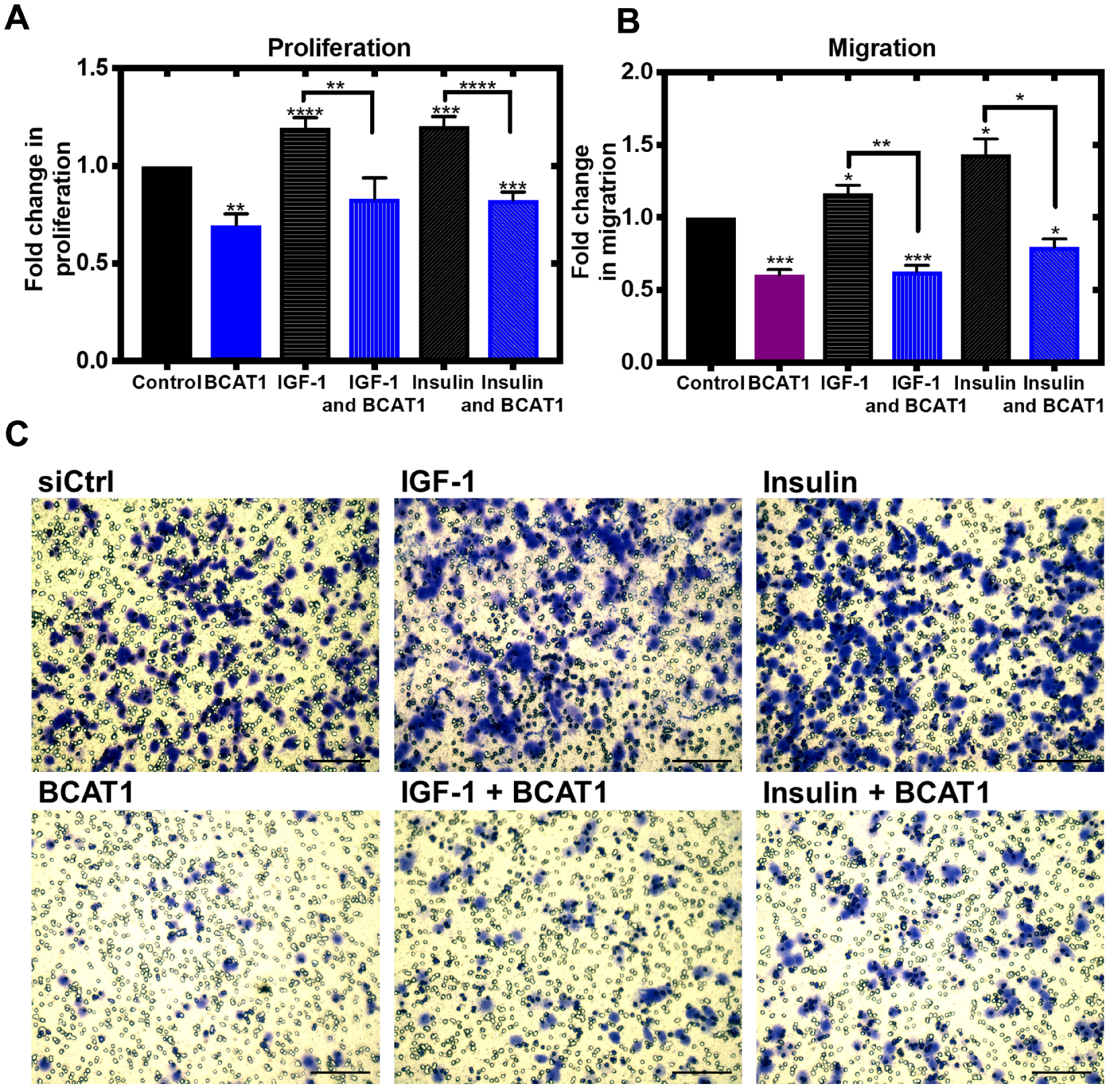
Knockdown of *BCAT1* significantly reduces insulin and IGF-1-mediated migration of MDA-MB-231 cells. Cells were treated with 20 nM *BCAT1* siRNA, 100 nM insulin and 100 ng/mL IGF-1 accordingly cell proliferation measured using the thymidine incorporation (TTI) assay and migration was assessed using cells seeded onto 8 μm Transwell inserts (Greiner Bio-One) coated with collagen and after 24 hours, migrated were fixed and stained with 0.2% Crystal Violet, solubilised and absorbance measured (**A**) Fold changes of mean values of disintegrations per minute (DPM) ± SEM, relative to control (**B**) Data presented as mean fold changes of absorbance at 590 nm. ^*^ *p* < 0.05, ^**^ *p* < 0.01 and ^***^ *p* < 0.001 (**C**) Representative images of migrated cells (scale bars = 100 μm).

We next showed that the IGF-1 and insulin-mediated increase in proliferation and migration of MDA-MB-231 cells was significantly attenuated by *BCAT1* knockdown indicating that BCATc controls proliferation and migration through the IGF-1 and insulin pathway (Figure 2A–2C). This was also observed in the SKOV-3, ovarian cell line (Supplementary Figure 1A–1D). The IGF-1/insulin pathway facilitates an orchestrated activation of numerous cell signalling events initiated through phosphorylation of insulin receptor substrates (IRS1/2) [23]. Numerous studies support a role for this pathway in tumorigenesis (reviewed in [24]) with overexpression of key proteins such as the IGF-1 receptor tyrosine kinase reported in breast cancer [25]. Leucine signalling is intrinsically linked with insulin with a suggestion that plasma BCAA levels play a role in insulin-mediated regulation through the Akt/mTOR pathway (as reviewed by [26]).

### BCATc significantly reduces IGF-1 mediated activation of ERK

**Figure 3:**
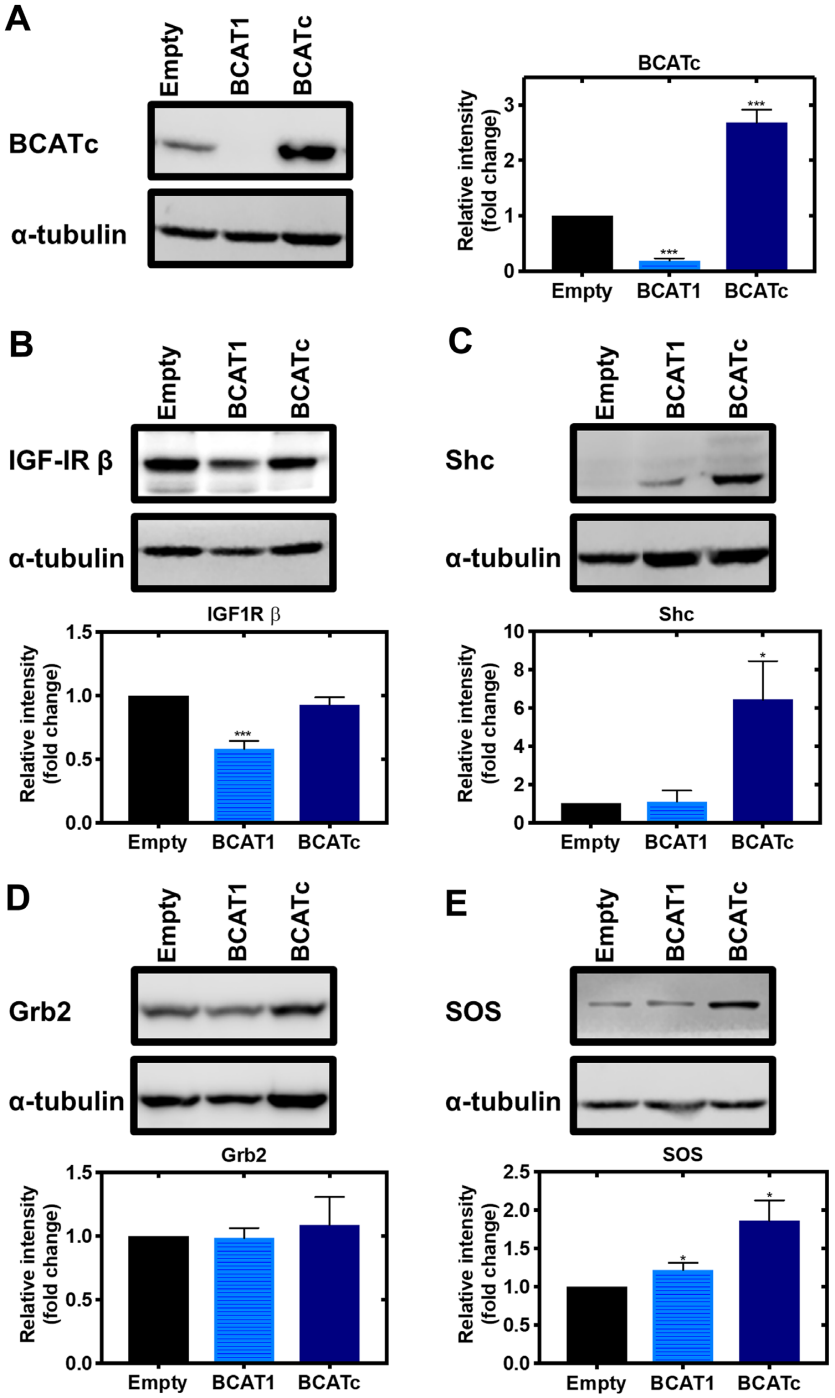
Knockdown and overexpression of *BCAT1* regulates IGF-1/insulin signalling cascade in MDA-MB-231 cells. Cells were treated with stable shRNA transfection for *BCAT1* knockdown (BCAT1) and overexpression (BCATc) for 8 days. (**A**) Western blot analysis confirmed *BCAT1* knockdown and overexpression. Western blot analysis was also used to assess changes in the protein expression of IGF-1 downstream targets (**B**) IGF-1Rβ (**C**) Shc (**D**) Grb2 (**E**) SOS. Respective densitometric analysis of fold changes of protein expression relative to ɑ-tubulin are presented. Data representative of mean ± SEM (*n* = 3) ^*^ *p* < 0.05, ^***^ *p* < 0.001 and ^****^ *p* < 0.0001.

Knockdown of BCATc led to a significant decrease in the levels of the IGF-1Rβ (Figure 3A and 3B). Elevated levels of BCAA have been reported to inhibit the activation of IGF-1R [27], indicating that BCATc upregulation together with BCAA, allows activation of the IGF-1R signalling cascade. Activation of the IGF-1/insulin pathway triggers two predominant signalling pathways, the PI3K/Akt and Ras/ERK pathway. ERK proteins are central transducers of extracellular signals from hormones, growth factors, cytokines and environmental stress; they regulate many cellular processes throughout the cell and phosphorylate transcription factors, cytoskeletal proteins and other protein kinases and enzymes [28–30].

**Figure 4:**
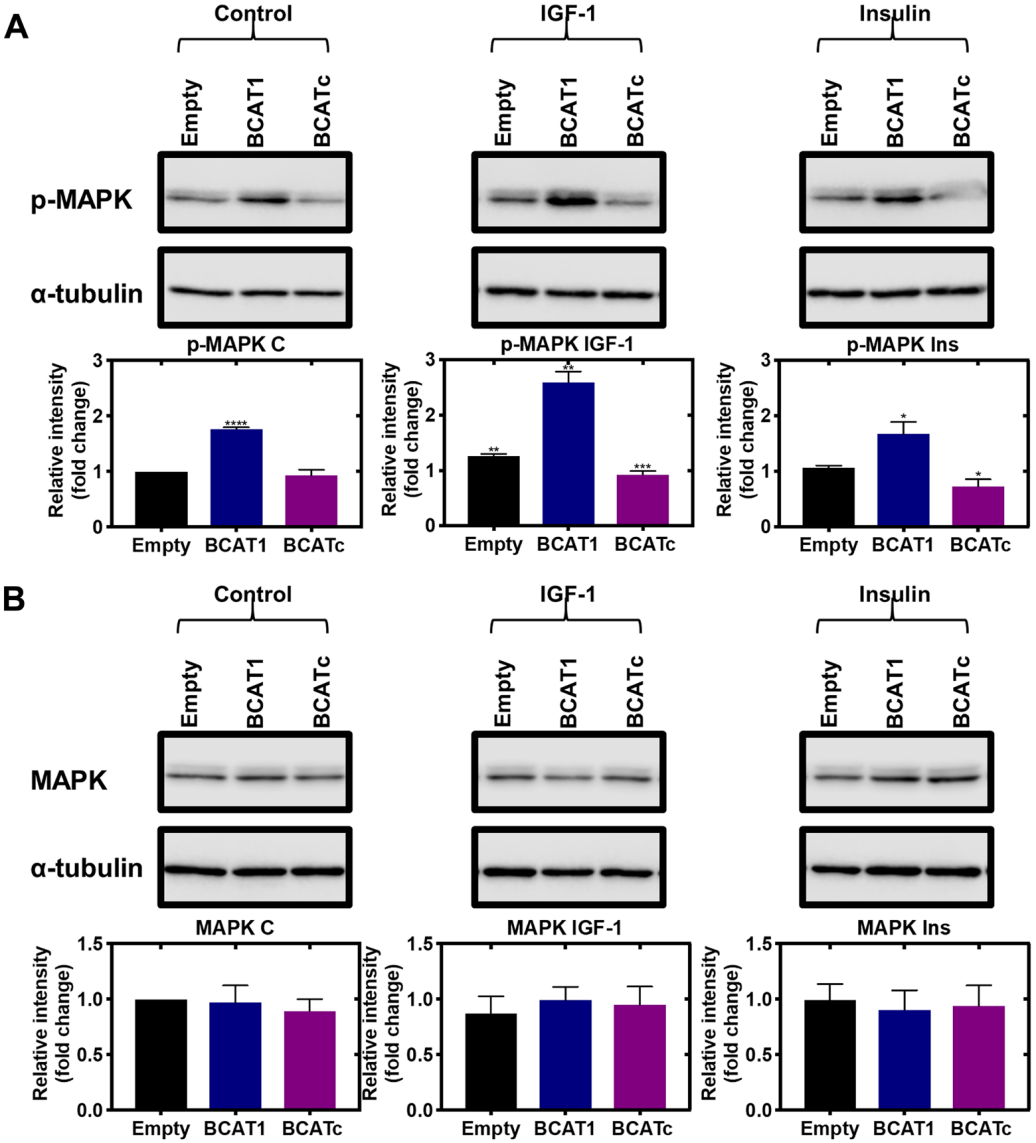
Knockdown and overexpression of *BCAT1* negatively regulates phosphorylation of MAPK in MDA-MB-231 cells. Cells were treated with stable shRNA transfection for *BCAT1* knockdown (BCAT1) and overexpression (BCATc) or unmodified pULTRA plasmid with no insertions as a control (Empty) for 8 days and treated with 100 nM insulin and 100 ng/mL IGF-1. (**A**) Western blot analysis was used to assess changes in the protein expression of phospho-MAPK and (**B**) MAPK. Respective densitometric analysis of fold changes of protein expression relative to ɑ-tubulin are presented below the immunoblots. Data representative of mean ± SEM (*n* = 3) ^*^ *p* < 0.05, ^**^ *p* < 0.01 and ^***^ *p* < 0.001.

**Figure 5:**
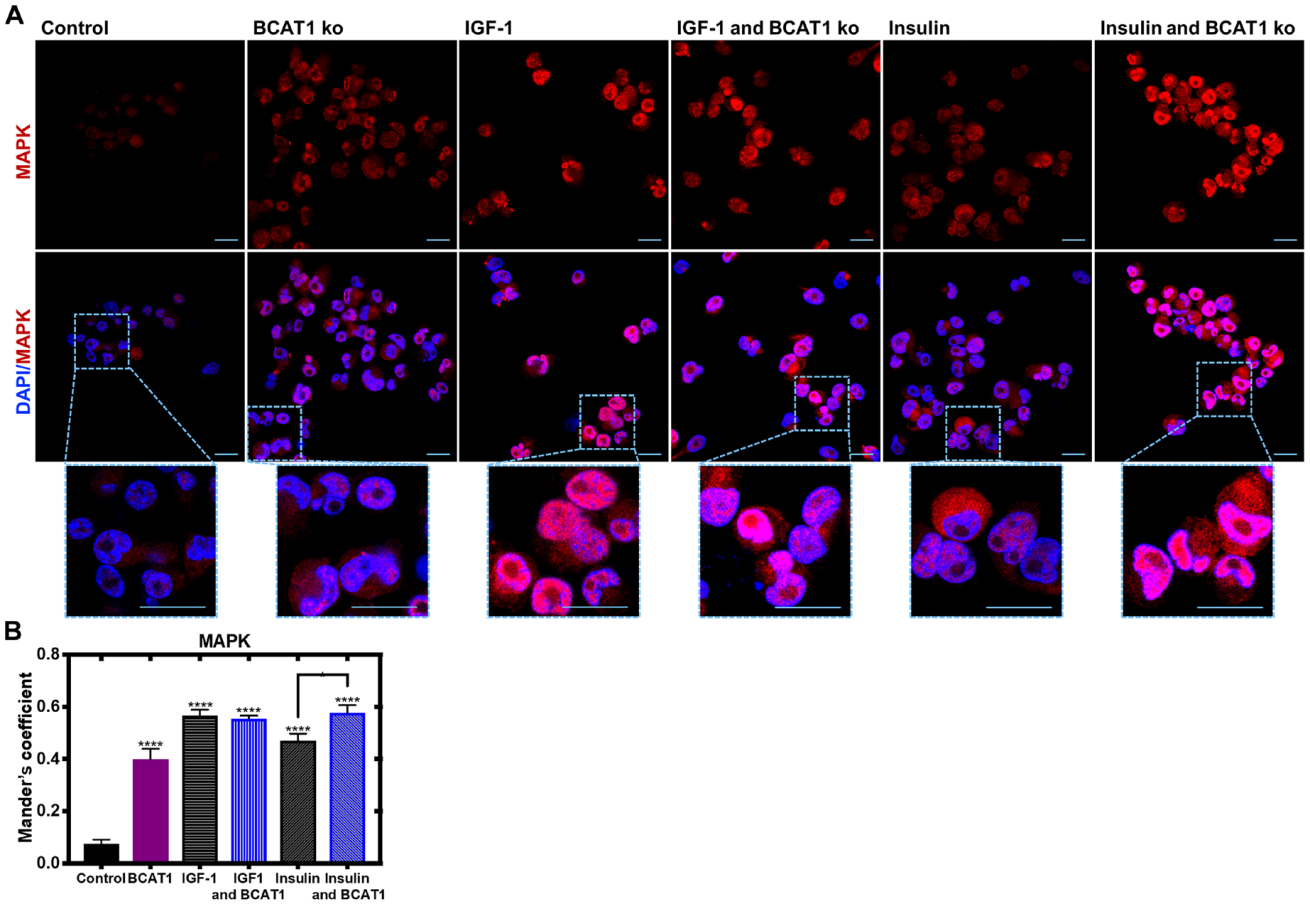
Insulin-mediated nuclear translocation of MAPK is significantly increased with BCAT1 knockdown in MDA-MB-231 cells. MDA-MB-231 cells were cultured on sterile glass coverslips (treated with poly-L-lysine) and treated with 20 nM *BCAT1* siRNA, 100 nM insulin and 100 ng/mL IGF-1. Cells were fixed with 4% paraformaldehyde and permeabilised with Triton ×. (**A**) Immunofluorescence Z stacked images of MDA-MB-231 cells probed with anti-MAPK (1:100) overnight at 4C, and then anti-rabbit Alexa Fluor^®^ 568 (1:250) for 1.5 hours. The coverslips were then mounted in VECTASHIELD HardSet antifade mounting medium with DAPI and imaged using a Leica SP8 confocal microscope. (**B**) Respective velocity analysis of Mander’s coefficient as compared to control. Data representative of mean ± SEM (*n* = 3) ^*^ *p* < 0.05 and ^***^ *p* < 0.001. Blue - DAPI; Red - MAPK.

Recruitment and phosphorylation of insulin receptor substrate 1/2 (IRS1/2) via IGF-1 acting on its receptor activates the Src homology 2 domain-containing (Shc) protein, which was shown to be significantly increased in response to BCATc overexpression (Figure 3C). The Shc substrate binds to the adaptor protein growth factor receptor-bound 2 (Grb2), levels of which were unaffected by BCATc and the associated guanine nucleotide exchange protein, son of sevenless (SOS), that we showed was increased when BCATc was overexpressed (Figure 3D and 3E, respectively). Interestingly, Grb2 has been assigned a regulatory role in the activation of the Ras/ERK pathway, through monomer/dimer interchange, that controls binding to SOS [31]. SOS allows the exchange of nucleotide guanosine diphosphate (GDP) bound to Ras with nucleotide guanosine triphosphate (GTP) in the cytosol. GTP-bound Ras allows membrane recruitment and activation of the serine/threonine protein kinase Raf to the plasma membrane [32]. Normally, this would enhance and activate mitogen-activated protein kinase (MAPK), which we expected to see in response to overexpression of BCAT. However, we show that knockdown of *BCAT1* increased phosphorylation of MAPK, even in the presence of IGF-1 or insulin and the opposite when BCATc was overexpressed (Figure 4A and 4B). Using confocal microscopy, we show that knockdown of *BCAT1* increased MAPK translocation to the nucleus indicating that BCATc may play an important role as a chaperone or scaffold protein for MAPK (Figure 5), a role previously reported for BCATc [33]. This response was significantly increased in response to insulin, which will affect downstream nuclear and cytoplasmic targets.

**Figure 6:**
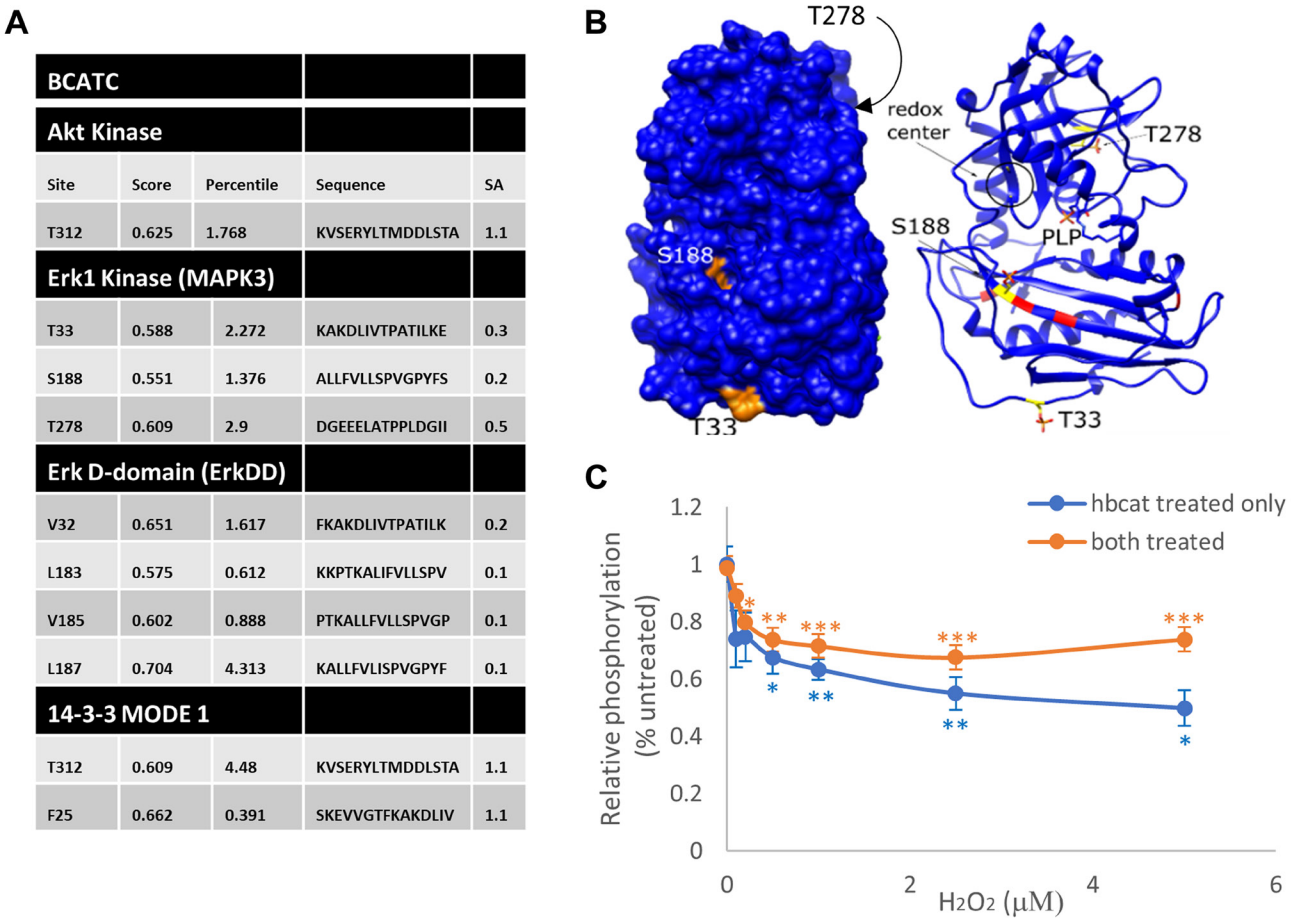
Putative phosphorylation sites for BCAT and its interaction with ERK (**A**) The BCAT protein sequence (accession #) was analysed using the Motif Scan program, Scansite 4 (http://scansite.mit.edu). Putative Akt and ERK1/2 phosphorylation sites on BCAT, as well as regions conforming to the ERK1/2 D-recognition site (DRS) substrate docking site (ERK-DD) and 14-3-3 phosphoamino-acid binding motif (14-3-3) are shown. (**B**) Phospho-Thr residues (TPO) and Phospho-Ser (SEP) were mapped onto the BCATc structure (PDB ID: BCAT) using UCSF Chimera. Putative phosphosites (S188, T33 and T278) are shown in the surface display (left) and ribbon display (right). The redox center of BCAT, which is composed of C335 and C338, is shown in the circle. Likewise, the predicted DRS binding site on BCATc is labelled red. (**C**) H_2_O_2_ titration curves of phosphorylation of BCATc by ERK2. ERK2 and BCATc were either both pre-treated with the indicated concentrations of H_2_O_2_ for 10 min at room temperature (orange) or only BCAT was pre-treated (blue). Excess H_2_O_2_ was then scavenged by catalase and the enzymes were combined in reaction buffer and incubated at 30°C for 30 min. The extent of phosphorylation was measured using the ADP-Glo assay. All activities were background corrected and normalized to that of the untreated controls. Error bars represent standard error (*n* = 6 for each dataset; ^*^ *p* < 0.05, ^**^ *p* < 0.01 and ^***^ *p* < 0.001 relative to the untreated control).

We have previously shown that BCATc is phosphorylated through redox-regulated PKC activation [16]. The BCAT proteins also have various phosphorylation motifs for MAPK defined using the Motif Scan program (http://scansite.mit.edu) (Figure 6A), highlighting 3 structurally accessible consensus sequences for MAPK, T33, S188 and T278 (Figure 6B). We show that BCATc is a substrate for MAPK phosphorylation where the redox sensor is important for phosphorylation. Interestingly, we show that when both proteins are oxidised then MAPK-mediated phosphorylation of BCATc is enhanced (Figure 6C). Therefore, we propose that the association between MAPK and BCATc is controlled through redox regulated phosphorylation governed by insulin and redox signalling, such that under conditions that promote tumour growth, BCATc levels not only increase but a change in function from transaminase to chaperone occurs. We evidence that BCATc shows both positive and negative regulation of key proteins involved in the Ras/ERK pathway with the ultimate decrease in IGF-mediated activation of ERK, suggesting that BCATc-induced regulation of cell proliferation and metastasis is unlikely to occur via this pathway.

### BCATc regulates cross-talk between the Ras/ERK and PI3K/Akt pathways

**Figure 7:**
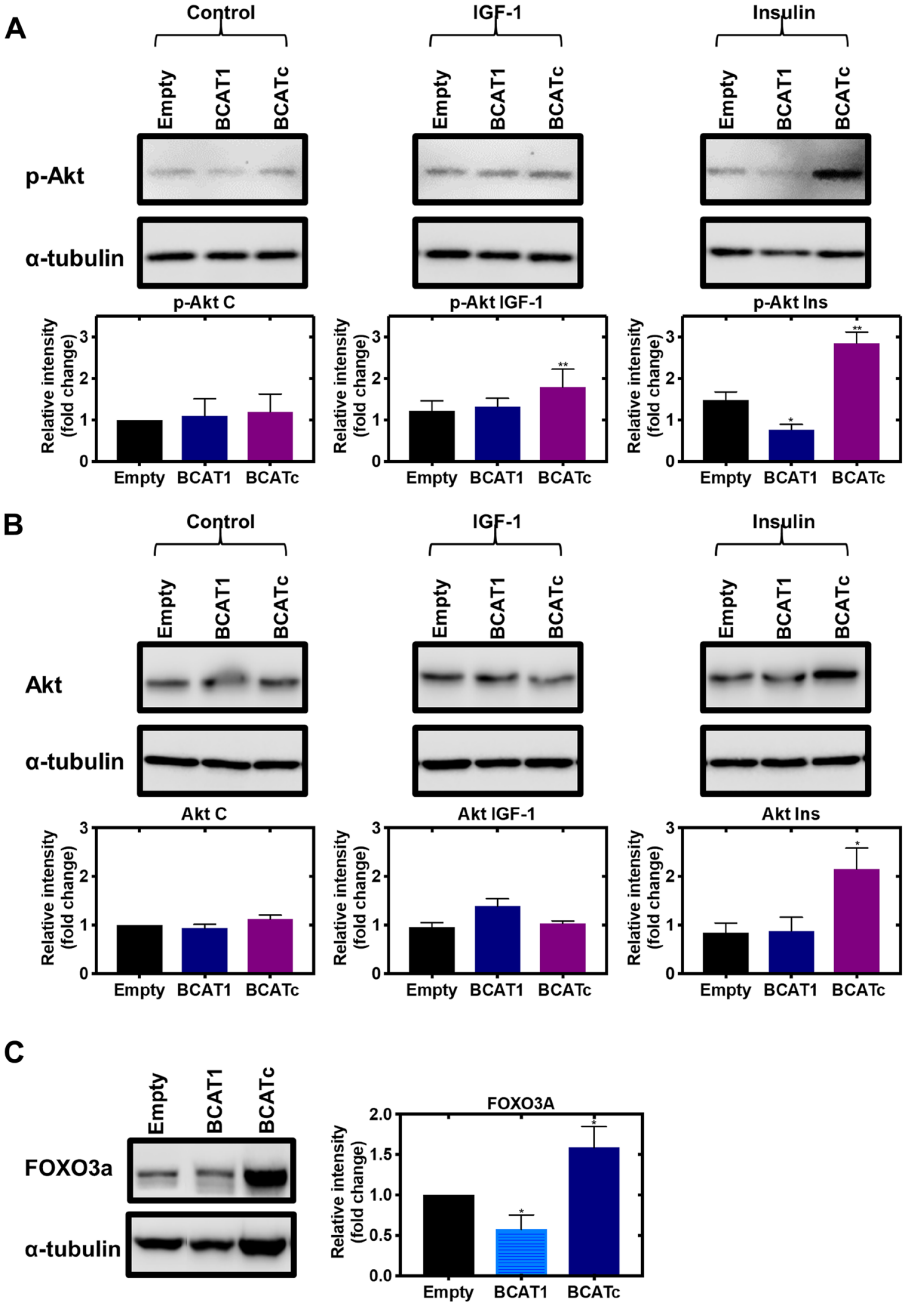
Knockdown and overexpression of *BCAT1* regulates the PI3K/Akt axis in MDA-MB-231 cells. Cells were treated with stable shRNA transfection for *BCAT1* knockdown (BCAT1) and overexpression (BCATc) or unmodified pULTRA plasmid with no insertions as a control (Empty) for 8 days and treated with 100 nM insulin and 100 ng/mL IGF-1. (**A**) Western blot analysis was used to assess changes in the protein expression of phospho-Akt (**B**) Akt and (**C**) FOXO3a. Respective densitometric analysis of fold changes of protein expression relative to ɑ-tubulin are presented below the immunoblots. Data representative of mean ± SEM (*n* = 3) ^*^ *p* < 0.05 and ^**^ *p* < 0.01.

Since increased activation of MAPK in response to *BCAT1* knockdown does not contribute to cell proliferation or migration, we next considered if these outcomes were through the PI3K/Akt signalling cascade. In response to IGF-1 and insulin signalling, overexpression of BCATc increased phosphorylation of Akt (Figure 7), whereas a significant increase in mammalian target of rapamycin (mTOR) was not reported (Supplementary Figure 2). Under normal cellular conditions, the metabolite α-ketoglutarate, the product of leucine transamination, activates mTOR. However, BCAT is regulated through phosphorylation and changes in the redox environment, which can prompt BCATc to associate with other signalling pathways, dependent on the stimuli, such as nutrient load, insulin/IGF-1 or changes in the redox environment [16]. Therefore, in response to IGF-1 or insulin the role of BCATc may switch from supporting mTOR activation to associate with proteins from the PI3K/Akt pathway. The dynamics and vectorality of these mechanisms are not entirely clear but more than likely will involve redox-regulated phosphorylation, such as that described for BCATc.

As discussed above, the levels of Shc and SOS were increased in response to increased BCATc expression (Figure 3C and 3D). Whilst it is accepted that the activation of IGF-1Rβ, Shc, Grb2 and SOS leads to MAPK activation [34, 35], SOS can also activate Ras [36]. It is known that Ras, which is activated with increased SOS, can also activate PI3K, Rac and Rho proteins associated with the regulation of the cytoskeleton and invasiveness of tumour cells [37]. Shc has been demonstrated to increase Grb2 interaction with PI3K on the p85 regulatory subunit leading to activation of the catalytic subunit of p110 [38, 39]. Moreover, Grb2 in a complex with the scaffolding protein Grb2-associated binder 1 (GAB) directly binds to p85 and enhances PI3K activation and subsequent Akt signalling [34, 40]. Thus, in this instance the increased level of these proteins may be associated with increased activation of the PI3K/Akt axis rather than increased activation of ERK.

**Figure 8:**
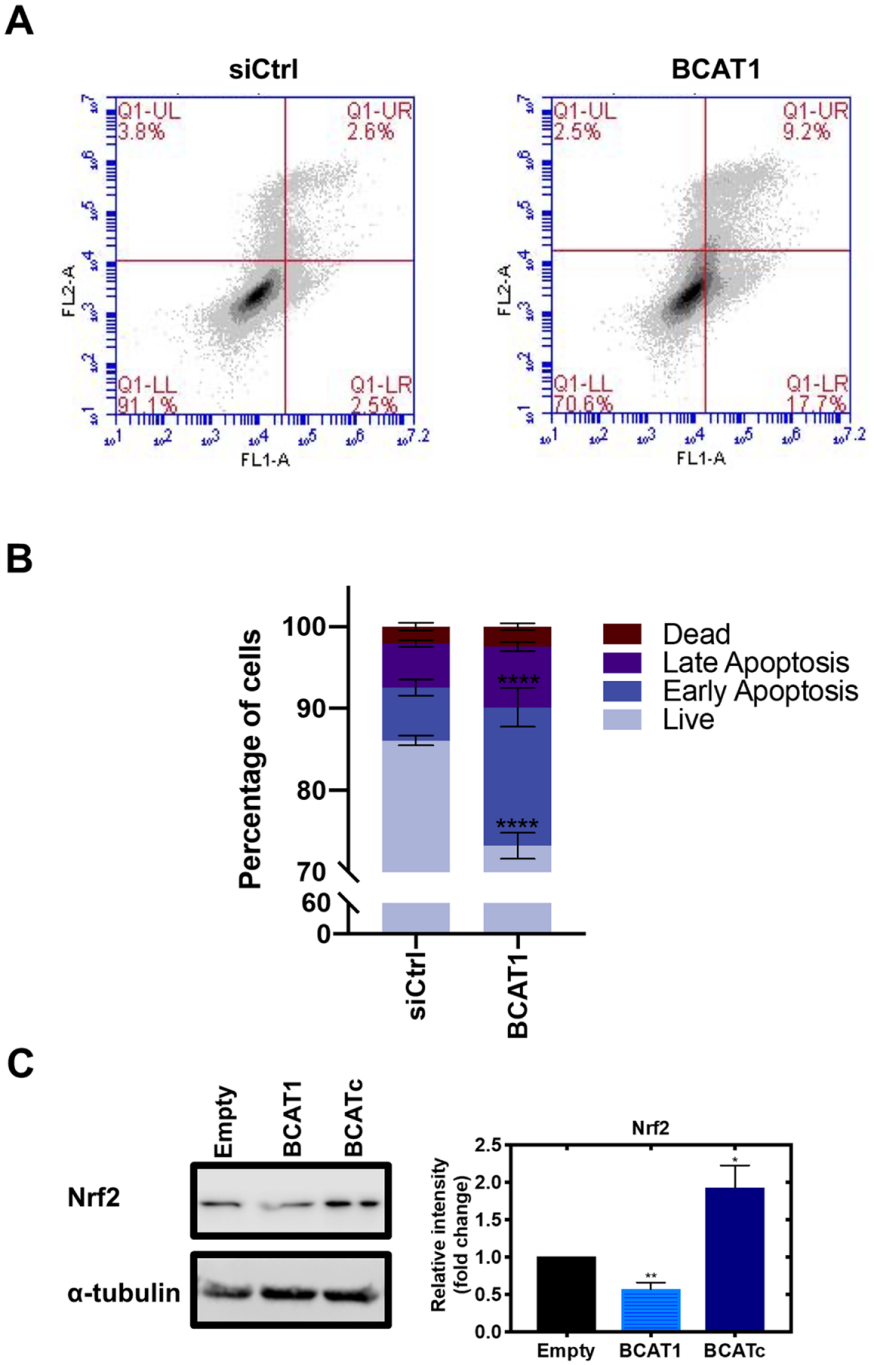
BCAT1 promotes tumour cell survival by evasion of apoptosis. Cells were treated with 20 nM *BCAT1* siRNA for 72 hours or transfection control, and cell apoptosis was determined by FITC-Annexin V and propidium iodide staining using flow cytometry. (**A**) Representative flow cytometric analysis (**B**) Data representative of mean ± SEM (*n* = 3) ^*^ *p* < 0.05 and ^**^ *p* < 0.01 (**C**) Western blot analysis was used to assess changes in the protein expression of Nrf2. Respective densitometric analysis of fold changes of protein expression relative to ɑ-tubulin are presented. Data representative of mean ± SEM (*n* = 3) ^*^ *p* < 0.05 and ^**^ *p* < 0.01.

Cross-talk of the RAS/ERK and PI3K/Akt pathways in the IGF-1/insulin signalling cascade allows tumour cells to utilise different effector molecules to promote tumour survival and progression (reviewed by [41]). One mechanism details that ERK phosphorylation of GAB1 inhibits GAB-1-mediated membrane recruitment of PI3K which in turn leads to the suppression of Akt signalling [42]. On the other hand, at high doses of IGF-1, Akt phosphorylates RAF at Ser259 and thus suppresses the activity of the RAS/ERK signalling pathway [43, 44], which may be related to the role of BCATc. Downstream of the PI3K/Akt pathway, we found that the transcription factor forkhead box O3 (FOXO3a) was upregulated in response to BCATc overexpression and knockdown of *BCAT1* led to reduced levels (Figure 7C). This effect was repeated in SKOV-3 cells (Supplementary Figure 3A). This member of the FOXO subfamily of forkhead transcription factors, which mediate a variety of cellular processes including cell cycle progression and apoptosis [45]. The role of FOXO3a in breast cancer has been suggested to be ERα+-dependent whereby FOXO3a upregulation in ERα+ MCF-7 cells reduced cell migration, however, in TNBC MDA-MB-231 cells FOXO3a was found to promote tumour cell migration [46, 47]. FOXO3a can act on cyclin-dependent kinase inhibitor 1B (p27^Kip1^) directly by disrupting cyclin D/CDK4 and cyclin E/CDK2 complexes to promote cell proliferation [48]. Therefore, positive regulation of this transcription factor by BCATc in TNBC may contribute to BCATc-mediated proliferation, migration and invasion. IGF-1R signalling and subsequent Akt phosphorylation of FOXO3a leads to its translocation from nucleus to cytoplasm, where it associates with 14.3.3 protein [49]. The 14.3.3 proteins act as scaffolds to integrate signalling proteins with targets involved in biological processes, including cell cycle regulation [50]. Interestingly, BCATc also has predicted binding motifs for Akt and 14-3-3 phosphorylation (Figure 6A), which like ERK may be the site of regulation for the interaction of these proteins. Overexpression of 14-3-3z has been associated with breast cancer recurrence indicating a role in therapy resistance, mediated via the downregulation of the pro-apoptotic proteins Bcl-2-associated death promoter (BAD) and Bcl-2-like protein (BIM) [51, 52]. Knockdown of *BCAT1* was found to increase the percentage of cells in the early apoptotic stage and reduce the percentage of live cells (Figure 8A and 8B), indicating that BCATc expression allows TNBC cells to evade apoptosis. The underlying mechanism by which hBCATc regulates cell apoptosis has not yet been elucidated. Hence, regulation of these Akt downstream effector proteins indicates an intrinsic role for BCATc for tumour progression mediated by cell proliferation, migration, cell cycle control and evasion of apoptosis through the regulation of FOXO3a.

### BCATc regulates cellular redox

We were interested to further elucidate the role of BCATc for tumour cell survival as increased glutamate levels contribute to glutathione (GSH) biosynthesis by facilitating the uptake of cystine via the xc-cysteine transporter, which is coupled to the efflux of glutamate [53]. GSH plays an important role in redox homeostasis and tumour cell survival by protecting cells from damage caused by reactive oxygen species (ROS) generated during oxidative stress [19]. Redox status is predominantly regulated by the transcription factor nuclear factor erythroid 2-related factor 2 (Nrf2) and its repressor protein kelch-like ECH-associated protein 1 (Keap1) in response to redox cellular status [54]. PI3K/Akt activation is essential for the nuclear translocation of Nrf2, whereby Akt inhibitors, but not MAPK inhibitors, were found to reduce Nrf2 transcriptional activation of antioxidant genes [55]. Akt can also increase the stability of Nrf2 by activating p21 which disrupts the interaction between Keap1 and Nrf2, reducing Nrf2 phosphorylation, thereby preventing its nuclear export and ubiquitination [56]. Under low concentrations of ROS, Nrf2 is bound to the E3 ubiquitin ligase Keap1 in the cytosol and degraded by the proteasome [57]. In response to oxidative stress, this interaction is inhibited, by the oxidation of cysteine residues on Keap1, thus allowing Nrf2 translocation to the nucleus to induce the expression of antioxidant genes such as the oxidoreductases, thioredoxin (Trx) and glutaredoxin (Grx) [58]. Here, levels of Nrf2 were increased with BCATc overexpression and decreased with knockdown of *BCAT1* (Figure 8C), suggesting that levels of BCATc positively regulate Nrf2 expression. Knockdown of BCAT1 also showed a decreased expression of Nrf2 in SKOV-3 cells, which resulted in an increase in ROS (Supplementary Figure 3B). Nrf2 modulates the expression of oxidoreductases, thioredoxin (Trx) and glutaredoxin (Grx) which are important in regulating cellular redox state [58]. Trx is a pro-oxidant with anti-apoptotic effects, including inactivation of caspase-3 [59], a primary player in the apoptotic signalling pathway, via a redox reactive nitrosothiol transfer [60]. Similarly, Grx1 inhibits apoptosis through Akt activation [61]. Therefore, whilst the role of BCATc mediates tumour survival by activating IGF-1/insulin PI3K/AKT signalling pathway, its expression may also influence Nrf2 regulation of the cellular redox state.

### Summary

IGF-1 is secreted by the liver in response to growth hormone, and its circulating levels remain constant via its unique interaction with its IGF binding proteins (IGFBPs). Unlike insulin, IGFs are also made in most cells of the body, where they play a key role in growth, survival and metabolism and are upregulated during cancer development. Insulin and IGF-1 are intricately linked systemically, for example during an insulin-resistant state the usual normalising processes are inhibited, leading to increased levels of circulating insulin and glucose. It also leads to a stimulation of hepatic IGF-1 synthesis, and downregulation of IGFBP-1 and IGFBP-2, resulting in an increased bioavailability of IGF-1. The high circulating insulin and IGFs, in an insulin-resistant state, is thought to be one of the mechanisms underlying the link between disturbed metabolism and cancer progression.

**Figure 9:**
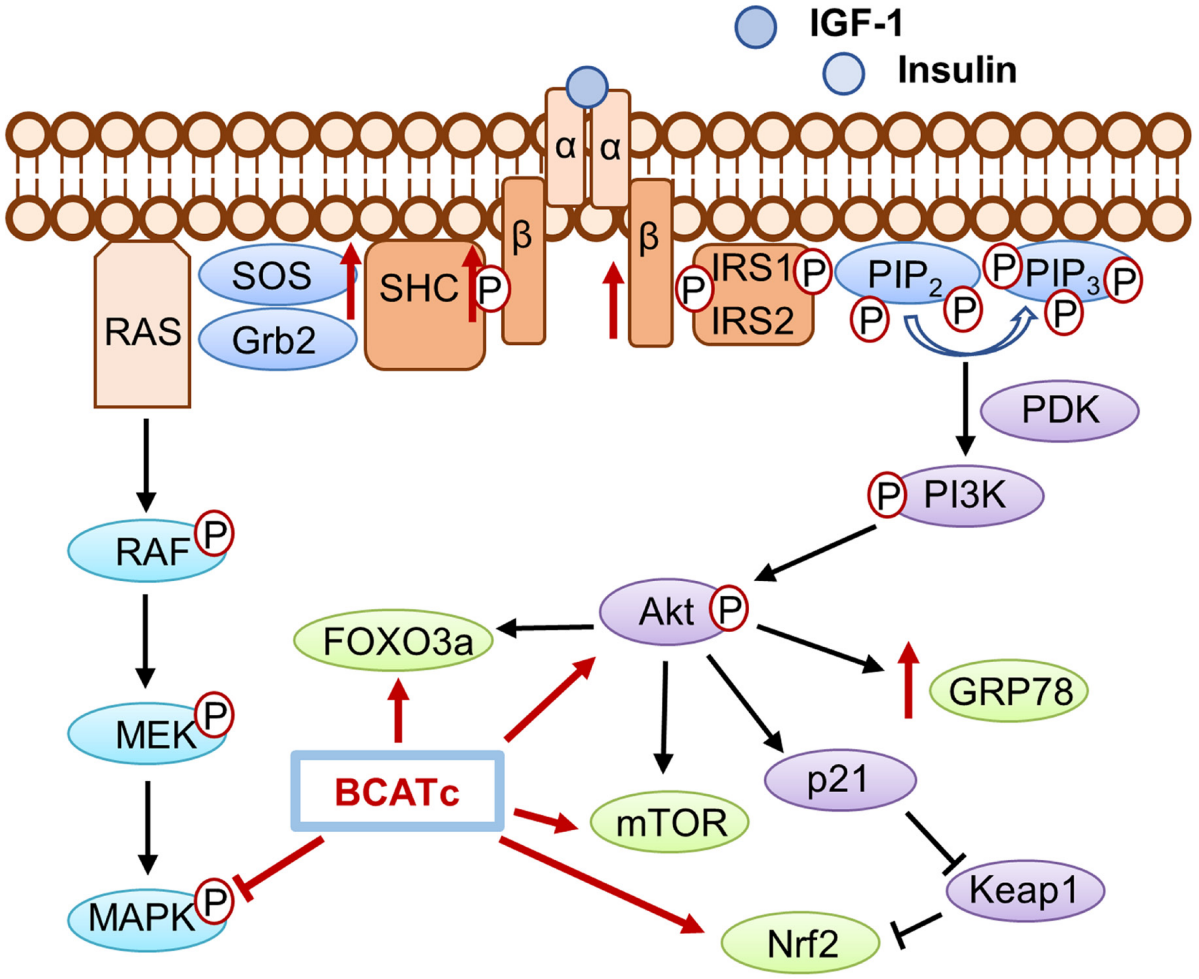
The role of BCATc on the insulin/IGF-1 signalling cascades. Activation of IGF-1R and insulin receptor, mediated by ligand binding of IGF-1 or insulin leads to the subsequent activation of the PI3K/AKT pathways and the Ras/MAPK pathways. Increased expression of BCATc increased levels of IGF-1Rβ, Shc and SOS however MAPK activation was increased with knockdown of *BCAT1* suggesting BCATc inhibits MAPK activation. Increased levels of hBCATc increased phosphorylation of Akt and the expression levels of the downstream effector proteins; FOXO3a, and Nrf2 thereby promoting cell proliferation, migration, and evasion of apoptosis in TNBC.

Deregulation of nutrient signalling that affects the PI3K/Akt and Ras/ERK pathways potentiates proliferation and metastasis of malignant cells including breast cancer [5]. Our findings suggest that BCATc behaves as a molecular chaperone or molecular scaffold that influences cell survival through the regulation and crosstalk of several key pathways. We have shown BCATc to increase activation of the IGF-1R signalling cascade with subsequent activation of the PI3K/Akt axis whilst subsequently down-regulating the RAS/ERK pathway. Moreover, BCATc promoted tumour cell survival through evasion of apoptosis mediated potentially through regulation of the redox status of the cells. In summary, BCATc provides TNBC cells with metabolic plasticity to alter dependence on the RAS/ERK and PI3K/Akt signalling cascades, in response to IGF-1/insulin, to promote tumour survival and progression (Figure 9).

## MATERIALS AND METHODS

### Contact of reagent and resource sharing

Further information and requests for resources and reagents should be directed to and will be fulfilled by the Lead Contact, Prof. Myra Conway (myra.conway@uwe.ac.uk).

### Experimental model and subject details

#### Cell lines and cell culture

MDA-MB-231 (triple negative breast cancer) were purchased from ATCC and cultured in Dulbecco’s Modified Eagle’s medium (DMEM) containing 10% foetal bovine serum (FBS) in a humidified incubator with 5% CO_2_ at 37°C.

### Method details

#### siRNA transfection

The siRNA sequences for *BCAT1* (Sense 5′CAUUAUCUACUGCUUCACAUU and antisense 5′ UGUGAAGCAGUAGAUAAUGUU) were previously designed and validated by Dr. Tom Forshaw (primers were synthesised by Eurofins Genomics, Germany). Cells were transfected with 20 nM *BCAT1* siRNA using Lipofectamine RNAimax or transfection solution prepared in Opti-MEM for 72 hours (Invitrogen, Paisley, UK).

#### Lentiviral shRNA transfection

The pULTRA-*BCAT1* plasmids were prepared. In brief, an H1 promoter-*BCAT1* shRNA sequence cassette was assembled in the pSUPER plasmid (VEC-PBS-0002, Oligoengine, Seattle, WA, a kind gift by Dr. Tim Craig) by ligation into the *BglII* and *XhoI* restriction sites. This was then amplified by PCR and the new plasmid assembled by ligation into the *SnaBI* restriction site of the pULTRA lentiviral vector (Addgene 24127, Cambridge, MA). For overexpression of *BCAT1,* the human gene was PCR amplified from our pET28a (Novagen, Madison, WI) bacterial overexpression vectors (Davoodi *et al.*, 1998) and the plasmid assembled by ligation into the *BamHI* and *EcoRI* restriction sites of pULTRA. Unmodified pULTRA plasmid with no insertions was included as a control. The pULTRA vector design included enhanced green fluorescent protein (GFP) expression, which was used to confirm uptake of the plasmid into transfected cells.

Lentiviral particles were produced by co-transfection of Sigma Mission lentiviral packaging mix to manufacturers guidelines and the respective pULTRA plasmid into HEK293T cells using the PEI method (PMC4246624) and supplementation of viral media with 1 mM sodium butyrate to enhance viral yield (PMC3830501/). The lentiviral particles were harvested 48 hours and 72 hours post-transfection of the HEK293T cells. For viral transfection of the MDA-MB-231 cells, harvested lentiviral particles were incubated with the cells for at least 8 days. To improve transfection efficiency 8 μg/mL polybrene was added to the shRNA transfected cells. Transfection efficiency was assessed by fluorescent microscopy as indicated by GFP activation.

### Western blot analysis

Cultured cells were lysed with radioimmune precipitation assay (RIPA) buffer supplemented with protease inhibitor and the protein concentration for each sample determined by Bradford assay. Aliquots of cell lysates (20 μg of protein) were resuspended in NuPAGE^®^ LDS (4% β-mercaptoethanol) and denatured at 95°C for 10 minutes. Proteins samples were separated on a 4–12% polyacrylamide gel and transferred onto activated (100% methanol) PVDF membrane. Membrane was blocked with 5% milk in TBST (50 mM Tris-HCl, 150 mM NaCl, 0.1% Tween-20) for 1 hour, washed with TBST, and incubated with appropriate primary antibodies overnight at 4°C. Next, membranes were washed with TBST and incubated with an appropriate secondary antibody at room temperature for 1 hour. Luminata Forte ECL solution was used to visualise the bands using the Li-Cor Odyssey system and densitometric analysis preformed using IMAGE STUDIO Lite software.

### ^3^H thymidine incorporation proliferation assay

Radioactively labelled (tritium) thymidine was used to measure the proliferation of MDA-MB-231 cells by the incorporation of [^3^H] thymidine into the DNA of dividing cells. Cells were seeded at a seeding density of 2 × 10^4^ cells in 24 well plates. Following treatment, cells were labelled with 0.1 μCi [^3^H] thymidine per well, incubated with 5% trichloroacetic acid at 4°C for 10 minutes, followed by a 1 hour incubation with 1 M sodium hydroxide. The resulting suspension was added to a vial containing 2 mL ultima gold liquid scintillation cocktail (Perkin Elmer Beaconsfield, Bucks, UK) and incorporated counts were measured using a Beckman Scintillation Counter LS6500. Data were recorded as disintegrations per minute (DPM).

### Migration and invasion assay

Thincert™ cell culture inserts (8 μm) (Greiner Bio-One) were coated with 10 μg/ml Collagen-I to measure migration and Matrigel^®^ to assess invasion 24 hours prior to cell seeding at room temperature. Cells were seeded in the upper chambers of the inserts at a density of 1 × 10^5^ MDA-MB-231 cells/mL in serum-free DMEM and 10% FBS DMEM placed in the lower chambers, in a humidified incubator at 37°C with 5% CO_2_. Following 24 hours, cells on the upper surface of the membrane were removed using an ethanol coated cotton swab and cells on the lower chamber fixed in 4% paraformaldehyde and stained with 0.2% Crystal Violet (Sigma). For each insert, representative images in 3 evenly distributed ×40 fields of view were captured using a light microscope (Zeiss AX10). To quantify migration and invasion of cells, 0.1% SDS in PBS was added to the lower chambers for 1 hour at room temperature and absorbance intensity measured on a plate reader at an excitation of 590 nm.

### ERK2 activation and purification

Activated doubly-phosphorylated MAPK1/ERK2 (pERK2) was generated and purified essentially as described by [62]. Briefly, 5× ATP solution (50 mM HEPES-KOH pH 7.38, 100 mM MgCl_2_, 20 mM DTT, 20 mM ATP) and dilution buffer (10 mM HEPES-KOH pH 7.38, 0.1% BME, 0.01% Triton X-100) were combined in a 1:4 ratio for a final volume of 25 mL. Inactive ERK2 and constitutively active MEK1 were then added to the solution before the reaction mix was divided into 0.5 mL aliquots. Reactions were then incubated at 30°C for 60 min. The solution was then dialyzed against ERK2 dialysis buffer (20 mM Tris-HCl, pH 8, 1 mM DTT) at 4°C overnight. Dialyzed ERK2 was purified by 1 mL HiTrap Q HP anion exchange column (GE Healthcare) that was preequilibrated with equilibration buffer (20 mM Tris-HCl, pH 8.0, 1 mM DTT, 0.1 M KCl, 10% glycerol). The reaction mix was then loaded onto the column and the column was washed with buffer A (20 mM Tris-HCl (pH 8.0), 1 mM DTT, 10% glycerol) with a step gradient of buffer A supplemented with 0 to 0.3 M KCl. Immobilized pERK2 eluted at about 0.2 M KCl. To assess the purity of the products, each fraction was resolved on a 5/10% SDS-PAGE gel and stained with Simply Blue Coomasie reagent. To determine which elution fractions contained the most protein, a Bradford total protein assay was completed. Briefly, elution fractions were assayed on a 96-well plate and incubated with Coomassie brilliant blue G-250 dye for 10 min at room temperature. Absorbance (595 nm) was measured using a TECAN Infinite F500 Pro microplate reader and negative control readings were subtracted from all readings to obtain background-corrected values. Likewise, the activity of each pERK2 fraction was determined by *in vitro* kinase assays, as described below, using the modular ERK2 peptide substrate, Sub-D. Fractions containing active pERK2 were pooled and then concentrated using an Amicon Ultra-15 (30K MWCO) before being dialyzed into ERK2 storage buffer (20 mM Tris-HCl, pH 8.0, 1 mM DTT, 1 mM EDTA, 0.2 M KCl, 10% glycerol) overnight at 4°C.

### H_2_O_2_-dependent oxidation of pERK2 and BCAT

pERK2 and the BCAT isoforms was diluted to concentrations of 465 nM and 2 μM, respectively. The proteins were then treated with various concentrations of H_2_O_2_ (0–5 μM) and incubated for 10 minutes at room temperature. Untreated controls were incubated with diH_2_O in place of H_2_O_2_. The remaining H_2_O_2_ was then scavenged by one-minute exposure to catalase (2.5 units). Next, 2.5 μL of pERK2 (treated and untreated) was incubated with 2.5 μL 4× kinase reaction buffer (8 mM MOPS, pH 7.2, 4 mM Beta-glyc-phosphate, 20 μM ATP, 8 mM MgCl_2_, 1.6 mM EGTA, 0.64 mM EDTA, 64 ng/μL BSA) and 5 μL of BCAT substrate (treated and untreated) at 30°C for 30 minutes. Negative control reactions contained 2.5 μL of diH_2_O in place of active ERK2. Following the 30°C incubation, the ERK2 selective inhibitor, SCH772984, was added to the reaction to quench the reaction. The effects of H_2_O_2_ on pERK2’s ability to phosphorylate the BCAT isoforms was then detected using the ADP Glo^®^ chemiluminescent kinase assay (Promega, Madison, WI) according to the manufacturer’s protocol. Briefly, the *trans*-phosphorylation reaction (10 μL) was transferred into a 96-well plate (Greiner, solid white, low-binding assay plates) and 10 μL of ADP Glo reagent was added to each well. The reaction was then incubated at room temperature for 40 min to deplete all remaining ATP [63]. The ADP produced by the enzyme/substrate interaction was then converted to ATP by adding 20 μL/well of Kinase Detection Reagent to yield a total assay volume of 40 μL/well. After the addition of Kinase Detection Reagent, the total reaction was incubated for 40 min and luminescence was detected with the TECAN Infinite F500-PRO microplate reader. Untreated controls received TBS vehicle at the same volume.

### DCFDA – ROS assay

To evaluate the effect of BCAT1 on the generation of ROS, *BCAT1*-siRNA (20 nM) transfected SKOV-3 cells were seeded overnight. Here, cells were treated with 100 μL of 2× 30 μM DCFDA solution and incubated in the dark at 37°C for 45 minutes. The fluorescent intensity was then measured on a fluorescence plate reader at excitation of 485 nm and emission of 535 nm.

### Apoptosis assay

To determine the effect of *BCAT1* siRNA knockdown on cell apoptosis, MDA-MB-231 cells were seeded at a density of 1 × 10^6^ cells per T-25 flask and treated with 20 nM *BCAT1* siRNA for 72 hours. Cells were detached and washed twice with ice cold PBS and resuspended in 1× binding buffer. Annexin V-FITC (Biolegend, San Diego) and PI (Fisher-Scientific) were added into the binding buffer and incubated for 10 min at room temperature in the dark. Analysis was performed on a BD Accuri™ C6 Flow Cytometer (BD Biosciences) to identify the subpopulations of the apoptotic cells.

### Statistical analysis

All data were expressed as means ± standard error of the mean (SEM). Significance levels for comparisons between groups were determined with unpaired two-tailed Student’s *t-test* using GraphPad Prism 8 (GraphPad Software). *P value*s of < 0.05 were considered statistically significant. For Western blots, protein levels were normalised to α-tubulin and relative band density reported as a fold change to control.

## SUPPLEMENTARY MATERIALS



## Abbreviations

BCAT: branched-chain aminotransferase
BCATm: mitochondrial branched-chain aminotransferase
BCATc: cytosolic branched-chain aminotransferase
BCAA: branched-chain amino acids
BCKD complex: branched-chain α-keto dehydrogenase complex
HER2: human epidermal growth factor receptor-2
PR: progesterone receptor
TNBC: triple negative breast cancer
IGF-1: insulin-like growth factor
EGF: epidermal growth factor
PI3K: phosphatidyl 3-kinase
MAPK: mitogen-activated protein kinase
mTOR: mammalian target of rapamycin
mTOR: mammalian target of rapamycin complex 1
GCN2: general control nonderepressible 2 kinase
FOXO3a: forkhead box O3
EGFR: epithelial growth factor receptor
IRS1/2: insulin receptor substrates
GLUT1: glucose transporter 1
SHC: Src homology 2 domain-containing
Grb2: growth factor receptor-bound 2
SOS: son of sevenless
Nrf2: nuclear factor (erythroid-derived 2)-like 2
GDP: nucleotide guanosine diphosphate
GTP: nucleotide guanosine triphosphate
siRNA: small interfering RNA
shRNA: short hairpin RNA
TTI: tritiated thymidine incorporation
ROS: reactive oxygen species
Grx: glutaredoxin
Trx: thioredoxin
H_2_O_2_: hydrogen peroxide
PVDF: polyvinylidene difluoride
TCA: tricarboxylic acid
