# Supplementary material for: BCATc modulates crosstalk between the PI3K/Akt and the Ras/ERK pathway regulating proliferation in triple negative breast cancer
Source: Oncotarget. 2020 May 26;11(21):1971–87. doi: 10.18632/oncotarget.27607 (PMC7260123; doi:10.18632/oncotarget.27607)
Supplement: Supplementary file 1 [file oncotarget-11-1971-s001.pdf]

BCATc modulates crosstalk between the PI3K/Akt and the Ras/ERK pathway regulating proliferation in triple negative breast cancer

SUPPLEMENTARY MATERIALS

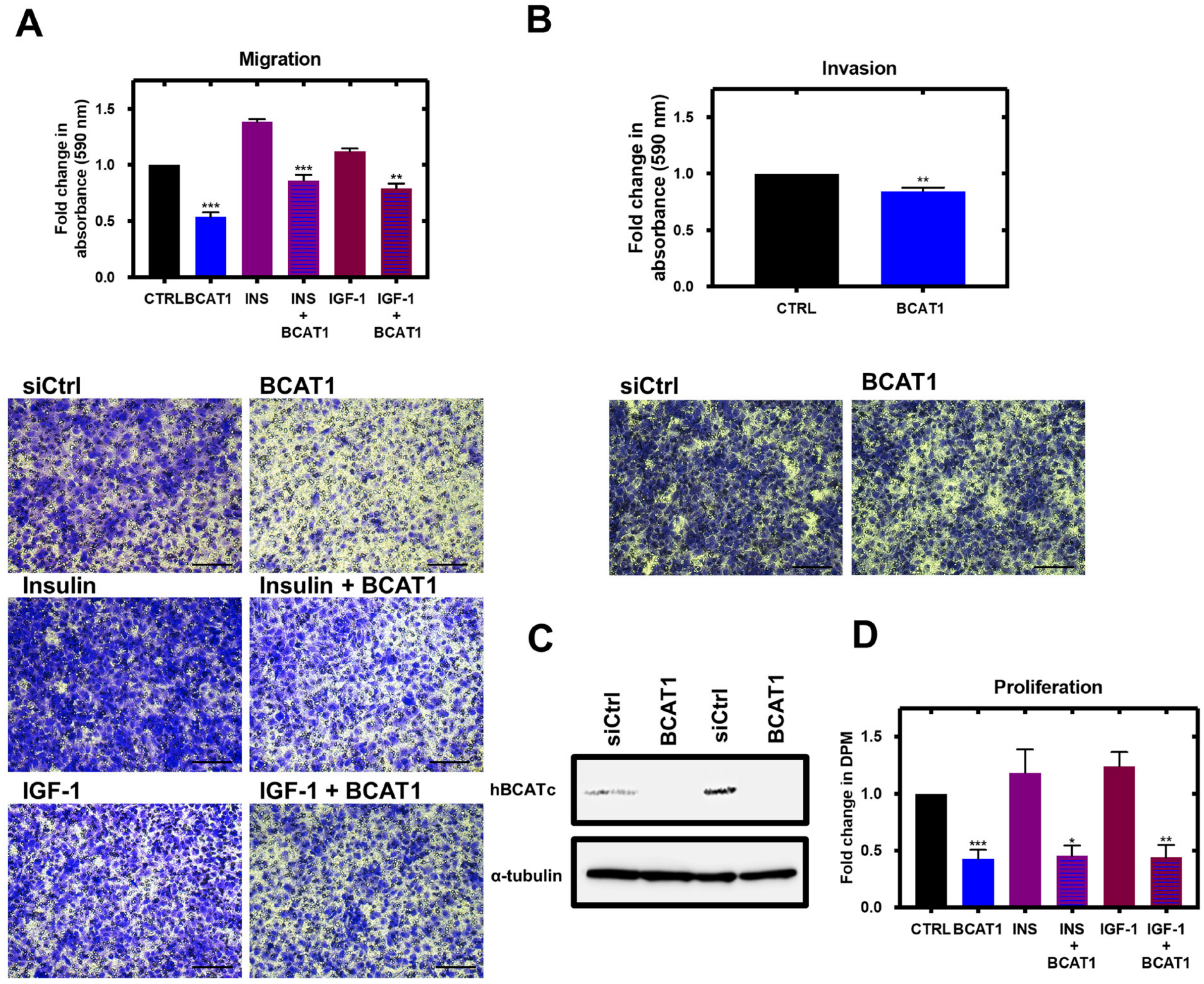

Supplementary Figure 1: Knockdown of *BCAT1* significantly reduces insulin and IGF-1-mediated migration of SKOV-3 cells. Cells were treated with 20 nM *BCAT1* siRNA, 100 nM insulin and 100 ng/mL IGF-1 accordingly cell proliferation measured using the thymidine incorporation (TTI) assay and migration was assessed using cells seeded onto 8 µm Transwell inserts (Greiner Bio-One) coated with collagen and after 24 hours, migrated were fixed and stained with 0.2% Crystal Violet, solubilised and absorbance measured (**A**) Migration (**B**) Invasion (**C**) Knockdown of BCATc (**D**) Proliferation data presented as mean fold changes of absorbance at 590 nm. $^{*}p < 0.05$, $^{**}p < 0.01$ and $^{***}p < 0.001$.

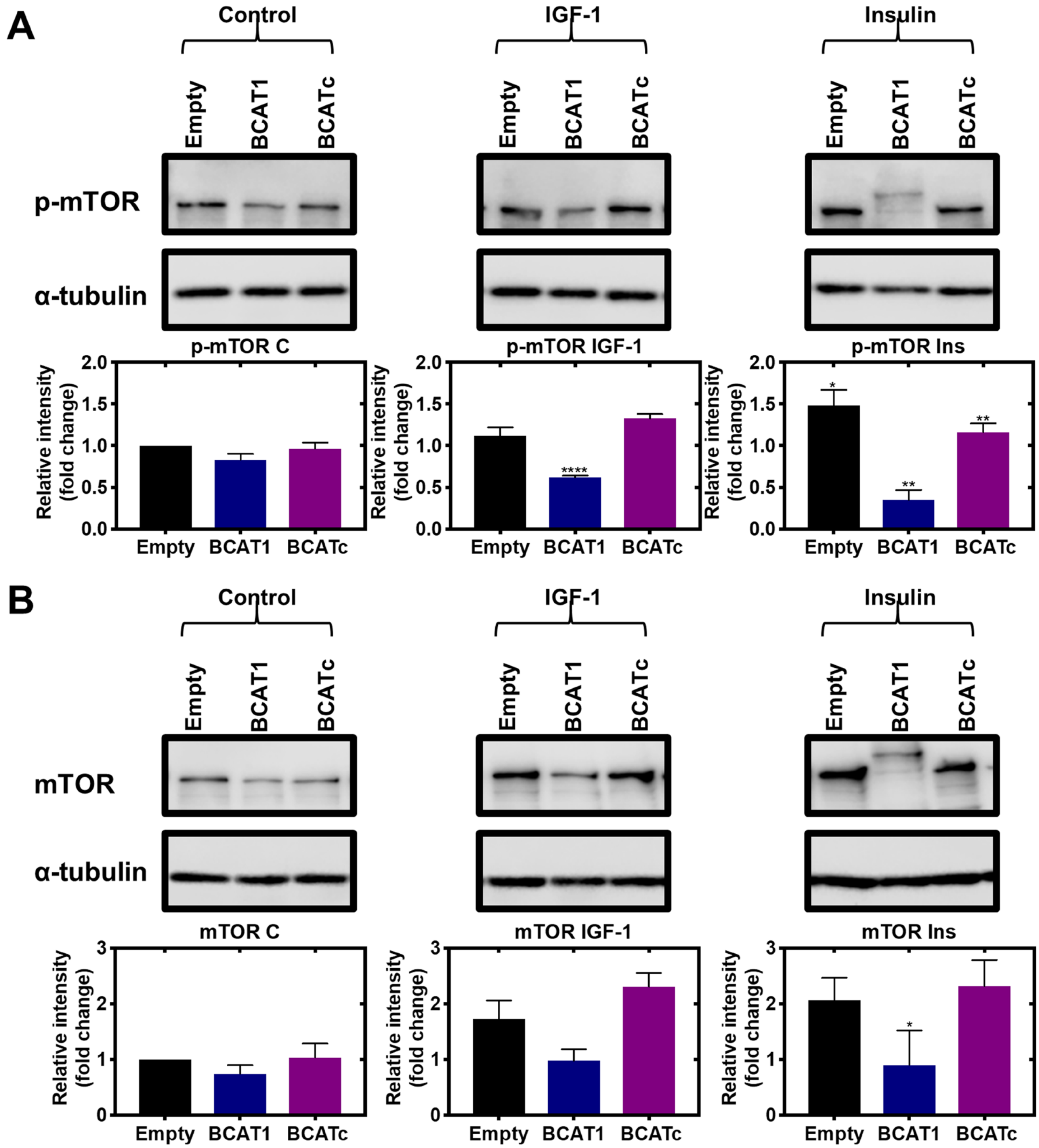

Supplementary Figure 2: Knockdown and overexpression of *BCAT1* regulates the mTOR axis in MDA-MB-231 cells. Cells were treated with stable shRNA transfection for *BCAT1* knockdown (BCAT1) and overexpression (hBCATc) or unmodified pULTRA plasmid with no insertions as a control (Empty) for 8 days and treated with 100 nM insulin and 100 ng/mL IGF-1. (**A**) Western blot analysis was used to assess changes in the protein expression of phospho-mTOR and (**B**) mTOR. Respective densitometric analysis of fold changes of protein expression relative to α-tubulin are presented below the respective immunoblots. Data representative of mean ± SEM (n = 3) $^{*}p < 0.05$, $^{**}p < 0.01$ and $^{****}p < 0.0001$.

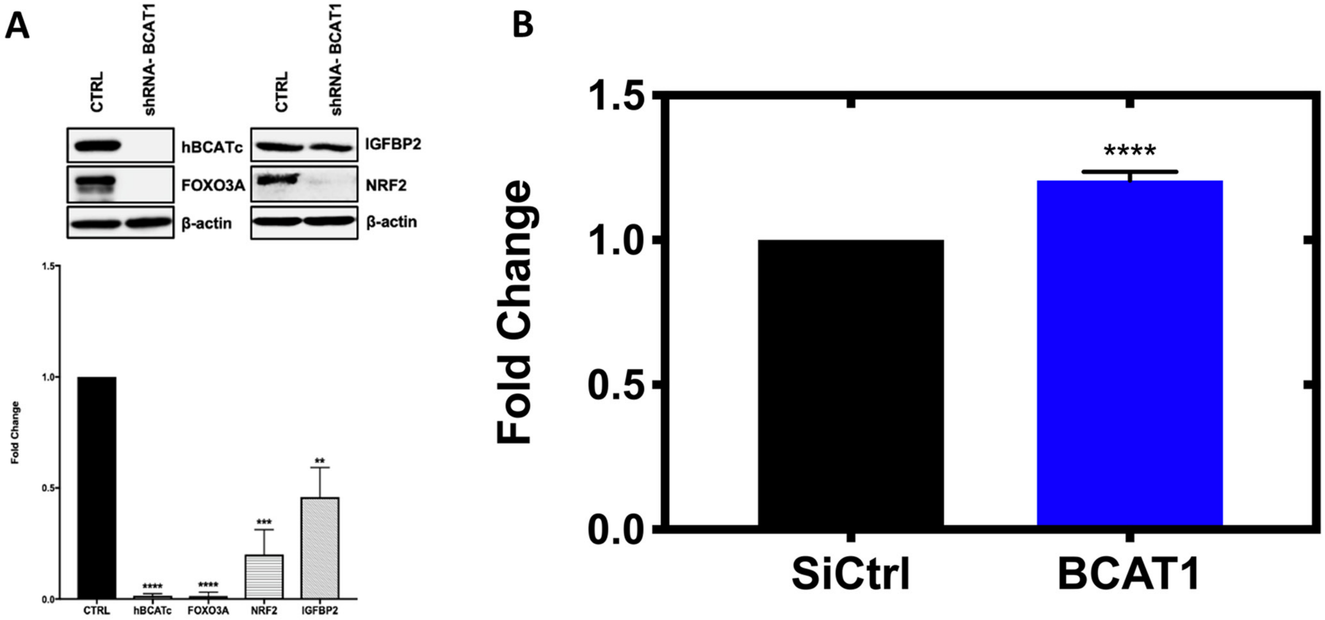

Supplementary Figure 3: Knockdown of *BCAT1* significantly reduces downstream signal of Akt signaling in SKOV-3 cells. Cells were treated with 20 nM *BCAT1* siRNA, Knockdown of BCATc (**A**) Western blot analysis of FOXO3A, Nrf2 relative to knockdown of BCAT1. (**B**) Changes in ROS generation were assessed using DCFDA assay as described in Materials and Methods. Data presented as mean fold changes of absorbance at 500 nm. $^{*}p < 0.05$, $^{**}p < 0.01$ and $^{***}p < 0.001$.
